# An integrated pipeline for next generation sequencing and annotation of the complete mitochondrial genome of the giant intestinal fluke, *Fasciolopsis buski* (Lankester, 1857) Looss, 1899

**DOI:** 10.7717/peerj.207

**Published:** 2013-11-12

**Authors:** Devendra Kumar Biswal, Sudeep Ghatani, Jollin A. Shylla, Ranjana Sahu, Nandita Mullapudi, Alok Bhattacharya, Veena Tandon

**Affiliations:** 1 Bioinformatics Centre, North-Eastern Hill University, Shillong, Meghalaya, India; 2 Department of Zoology, North-Eastern Hill University, Shillong, Meghalaya, India; 3 M/s Genotypic Technologies, Bangalore, India; 4 School of Life Sciences, Jawaharlal Nehru University, New Delhi, India

**Keywords:** *Fasciolopsis buski*, Mitochondria, Next generation sequencing, Contigs

## Abstract

Helminths include both parasitic nematodes (roundworms) and platyhelminths (trematode and cestode flatworms) that are abundant, and are of clinical importance. The genetic characterization of parasitic flatworms using advanced molecular tools is central to the diagnosis and control of infections. Although the nuclear genome houses suitable genetic markers (e.g., in ribosomal (r) DNA) for species identification and molecular characterization, the mitochondrial (mt) genome consistently provides a rich source of novel markers for informative systematics and epidemiological studies. In the last decade, there have been some important advances in mtDNA genomics of helminths, especially lung flukes, liver flukes and intestinal flukes. *Fasciolopsis buski*, often called the giant intestinal fluke, is one of the largest digenean trematodes infecting humans and found primarily in Asia, in particular the Indian subcontinent. Next-generation sequencing (NGS) technologies now provide opportunities for high throughput sequencing, assembly and annotation within a short span of time. Herein, we describe a high-throughput sequencing and bioinformatics pipeline for mt genomics for *F. buski* that emphasizes the utility of short read NGS platforms such as Ion Torrent and Illumina in successfully sequencing and assembling the mt genome using innovative approaches for PCR primer design as well as assembly. We took advantage of our NGS whole genome sequence data (unpublished so far) for *F. buski* and its comparison with available data for the *Fasciola hepatica* mtDNA as the reference genome for design of precise and specific primers for amplification of mt genome sequences from *F. buski*. A long-range PCR was carried out to create an NGS library enriched in mt DNA sequences. Two different NGS platforms were employed for complete sequencing, assembly and annotation of the *F. buski* mt genome. The complete mt genome sequences of the intestinal fluke comprise 14,118 bp and is thus the shortest trematode mitochondrial genome sequenced to date. The noncoding control regions are separated into two parts by the tRNA-Gly gene and don’t contain either tandem repeats or secondary structures, which are typical for trematode control regions. The gene content and arrangement are identical to that of *F. hepatica*. The *F. buski* mtDNA genome has a close resemblance with *F. hepatica* and has a similar gene order tallying with that of other trematodes. The mtDNA for the intestinal fluke is reported herein for the first time by our group that would help investigate Fasciolidae taxonomy and systematics with the aid of mtDNA NGS data. More so, it would serve as a resource for comparative mitochondrial genomics and systematic studies of trematode parasites.

## Introduction

*Fasciolopsis buski*, often called the giant intestinal fluke, is one of the largest digenean trematode flatworms infecting humans and found primarily in Asia and the Indian subcontinent, also occurring in Taiwan, Thailand, Laos, Bangladesh, India, and Vietnam. The trematode predominates in areas where pigs are raised, they being the most important reservoirs for the organism and where underwater vegetables viz. water chestnut, lotus, caltrop and bamboo are consumed. It is an etiological agent of fasciolopsiasis, a disease that causes ulceration, haemorrhage and abscess of the intestinal wall, diarrhoea, and even death if not treated properly. Interestingly, most infections are asymptomatic with high rates of infection (up to 60%) in India and the mainland China (Le et al., 2004). Among animals, pigs are the main reservoir of *F. buski* infection. In India, the parasite has been reported from different regions including the Northeast and variations in the morphology of the fluke have been observed from different geographical regions (Roy & Tandon, 1993). *F. buski* occurs in places with warm, moist weather and is the only single species in the genus found in aquatic environments. The complex life cycle combined with the specific immune evasion traits of parasites make research and drug or vaccine programs for intestinal flukes very difficult; consequently, new methods to control this parasite are required. Being one of the most important intestinal flukes from an epidemiological point of view, *F. buski* seeks considerable attention from the scientific community and the available gene sequences for the organism on the public domain remain scarce thereby restricting research avenues. Therefore, fasciopsiasis has become a public health issue and is of major socioeconomic significance in endemic areas.

Metazoan mitochondrial (mt) genomes, ranging in size from 14 to 18 kb, are typically circular and usually encode 36–37 genes including 12–13 protein-coding genes, without introns and with short intergenic regions (Wolstenholme, 1992). Due to their maternal inheritance, faster evolutionary rate change, lack of recombination, and comparatively conserved genome structures mitochondrial DNA (mtDNA) sequences have been extensively used as molecular markers for studying the taxonomy, systematics, and population genetics of animals (Li et al., 2008; Catanese, Manchado & Infante, 2010). At the time of writing this manuscript, quite a number of complete metazoan mt genomes are already deposited in GenBank (Benson et al., 2005) and other public domain databases viz. Mitozoa (D’Onorio de Meo et al., 2011), mainly for Arthropoda, Mollusca, Platyhelminthes, Nematoda, and Chordata (Chen et al., 2009). Presently, the class Trematoda comprises about 18,000 nominal species, and the majority of them can parasitize mammals including humans as their definitive host (Olson et al., 2003). Despite their medical and economical significance, most of them still remain poorly understood at the molecular level. In particular, the complete mt genomes of the species belonging to the family Fasciolidae are not at all available in the public domain. Complete or near-complete mt genomes are now available for 15 odd species or strains of parasitic flatworms belonging to the classes Trematoda and Cestoda. To date, a PCR-based molecular characterization using ITS1&2 molecular markers for *F. buski* have been carried out (Prasad et al., 2007). However, further datasets generated by high-throughput sequencing and comparative transcriptome analysis could bring a more comprehensive understanding of the parasite biology for studying parasite-host interactions and disease as well as parasite development and reproduction, with a view towards establishing new methods of prevention, treatment or control.

Until quite recently, sequencing of mt genomes was somewhat challenging and a daunting task. It has been approached using the conventional strategy of combining long-range PCR with subsequent primer walking. The paradigm shift caused by the third generation sequencing technologies have paved the way for Next-Generation Sequencing (NGS) technologies, which encourages proposals for more straightforward integrated pipelines for sequencing complete mt genomes (Jex, Littlewood & Gasser, 2010) that are more cost effective and less time consuming.

Here in, we present a straightforward approach for reconstructing novel mt genomes directly from NGS data generated from total genomic DNA extracts. We took advantage of the whole genome sequence data for *F. buski* (DK Biswal, S Ghatani, JA Shylla, R Sahu, N Mullapudi, A Bhattacharya, V Tandon, unpublished data), generated by NGS and its comparison with the existing data for the *F. hepatica* mt genome sequence to design precise and specific primers for amplification of mt genome sequences of *F. buski*. We then carried out long-range PCR to create a NGS library enriched in mt DNA sequences. We utilized two different next generation sequencing platforms to completely sequence the mitochondrial genome, and applied innovative approaches to assemble the mitochondrial genome *in silico* and annotate it. When verifying one region of the assembly by Sanger sequencing it was found to match our assembly results. The purpose of the present study was to sequence the mt genome of *F. buski* for the first time with a novel strategy, compare its sequences and gene organization, identify any adaptive mutations in the 12 protein-coding genes of the intestinal parasite species, and to reconstruct the phylogenetic relationships of several species of Trematoda and Cestoda in the Phylum Platyhelminthes, using mtDNA sequences available in GenBank.

## Materials & Methods

### Parasite material and DNA extraction

Live adult *F. buski* were obtained from the intestine of freshly slaughtered pig, *Sus scrofa domestica* at local abattoirs meant for normal meat consumption and not specifically for this design of study. The worms recovered from these hosts represented the geographical isolates from Shillong (coordinates 25.57°N 91.88°E) area in the state of Meghalaya, Northeast India. Eggs were obtained from mature adult flukes by squeezing between two glass slides. For the purpose of DNA extraction, adult flukes collected from different host animals were processed singly; eggs recovered from each of these specimens were also processed separately. The adult flukes were first immersed in digestion extraction buffer [containing 1% sodium dodecyl sulfate (SDS), 25 mg Proteinase K] at 37°C for overnight. DNA was then extracted from lysed individual worms by standard ethanol precipitation technique (Sambrook, Fitsch & Maniatis, 1989) and also extracted from the eggs on FTA cards using Whatman’s FTA Purification Reagent. DNA was subjected to a series of enzymatic reactions that repair frayed ends, phosphorylate the fragments, and add a single nucleotide ‘A’ overhang and ligate adaptors (Illumina’s TruSeq DNA sample preparation kit). Sample cleanup was done using Ampure XP SPRI beads. After ligation, ∼300–350 bp fragment for short insert libraries and ∼500–550 bp fragment for long insert libraries were size selected by gel electrophoresis, gel extracted and purified using Minelute columns (Qiagen). The libraries were amplified using 10 cycles of PCR for enrichment of adapter-ligated fragments. The prepared libraries were quantified using Nanodrop and validated for quality by running an aliquot on High Sensitivity Bioanalyzer Chip (Agilent). 2X KapaHiFiHotstart PCR ready mix (Kapa Biosystems Inc., Woburn, MA) reagent was used for PCR. The Ion torrent library was made using Ion Plus Fragment library preparation kit (Life Technologies, Carlsbad, US) and the Illumina library was constructed using TruSeqTM DNA Sample Preparation Kit (Illumina, Inc., US) reagents for library prep and TruSeq PE Cluster kit v2 along withTruSeq SBS kit v5 36 cycle sequencing kit (Illumina, Inc., US) for sequencing.

### Primer design strategy and Polymerase Chain Reaction (PCR)

**Figure 1:**
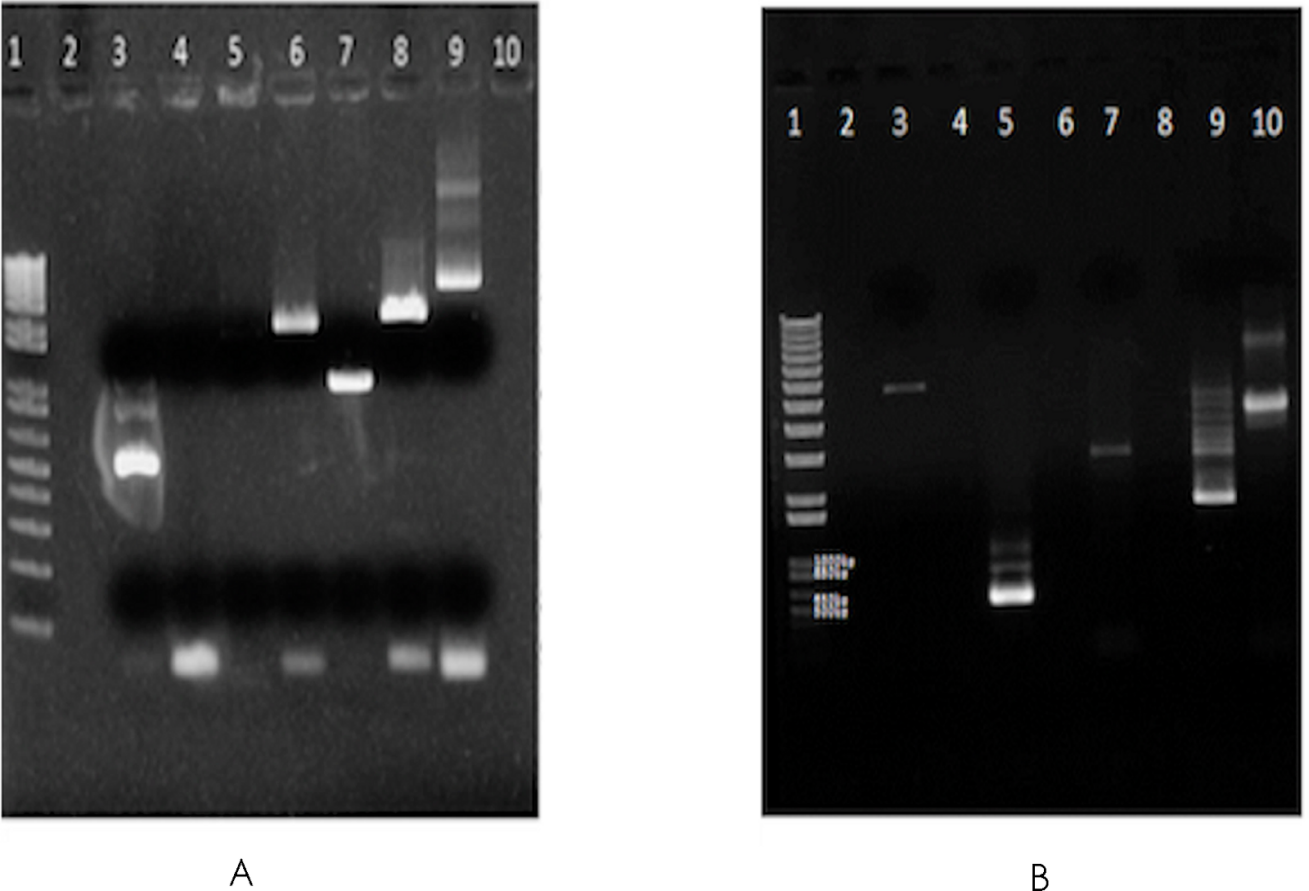
Gel images of the long range PCR products. Long-range PCR carried out using 10 ng of genomic DNA from *F. buski* (FD2 and FD3 samples). Gel-eluted bands corresponding to different products that were pooled for NGS library construction are shown. (A) SO_1625_mt gel (elution). Lane Order: Lane 1-1 kb plus ladder, Lane 2-Blank, Lane 3-P1 primer pair, Lane 4-P2 primer pair, Lane 5-P3 primer pair, Lane 6-P4 primer pair, Lane 7-P5 primer pair, Lane 8-P6 primer pair, Lane 9-P7 primer pair, Lane10-Blank. Lanes 3, 5, 6, 7, 8, and 9 (both bands) were taken for Ion Torrent Library Prep. (B) SO_1625_mt gel (elution). Lane Order: Lane 1-1 kb plus ladder, Lane 2-Blank, Lane 3-P7 + P2 primer pair, Lane 4-Blank, Lane 5-P1 primer pair, Lane 6-Blank, Lane 7-P2 primer pair, Lane 8-Blank, Lane 9-P3 primer pair, Lane 10-P7 primer pair. Lanes 3, 5 (all bands), 7, 9 (bright band) and Lane 10 (both bands) were taken for Ion Torrent Library Prep.

∼16 million 100 base-paired end reads were available for *F. buski* as a part of an independent attempt towards whole genome sequencing of *F. buski*. In order to recover mtDNA coding sequences from this data, *Fasciola hepatica* mt genome with accession AF216697.1 was retrieved from GenBank as a reference mt Genome and alignment using Bowtie (v2-2.0.0-beta6/bowtie2 –end-to-end –very-sensitive –no-mixed –phred64) (Langmead et al., 2009). In all, 1625 paired end reads were obtained, which were aligned to different intervals in the *F. hepatica* mt genome, covering ∼3 kb of the 14 kb *F. hepatica* mt genome. Accordingly, primers were designed at these regions, using sequence information from *F. buski* to ensure optimum primer designing as shown in Table 1. Long-range PCR was carried out using 10 ng of genomic DNA from *F. buski* and the following PCR conditions: 10 ng of FD-2 DNA with 10 µM Primer mix in 10 µl reaction PCR cycling conditions −98°C for 3 min, 35 cycles of 98°C for 30 s, 60 for 30 s, 72 for 2 min 30 s, final extension 72°C for 3 min and 4°C hold. The bands were gel-eluted corresponding to different products and pooled for NGS library construction (Fig. 1).

**Table 1:** Primer sequences used in the study.

Primer name	Primer sequence	Product	Expected length	Observed length (bp)
F1	TACATGCGGATCCTATGG	P1	1525	500, 700, 1000
F2	AAAGACATACAAACAACAAC			
F3	TCTTTAGTGTATTCTTTGGGTCATG	P2	2660	3000
F4	AACAACCCCAACCTACCCT			
F5	GTTTGTTGAGGGTAGGTTGGGG	P3	1623	1600
F6	CAAATCATTAATGCGAGG			
F7	CTTTTTGATGCCTGTGTTCATAG	P4	2010	2000
F8	ACCTTTCAAACAATCCCCCA			
F9	CGGATTTATAGATGGTAGTGCCTG	P5	1037	1000
F10	CCGGATATACACTAACAAACATAATTAAG			
F11	GTTTGTTAGTGTATATCCGGTTGAAG	P6	2361	2200
F12	GGCAGCAACCAAAGTAGAAGA			
F13	TATTTCTTGGTTGTTGGAGGCTAT	P7	3783	4000, 8000
F14	TCTATAGAACGCAACATAGCATAAAAG			

### NGS library construction, sequencing and assembly

**Figure 2:**
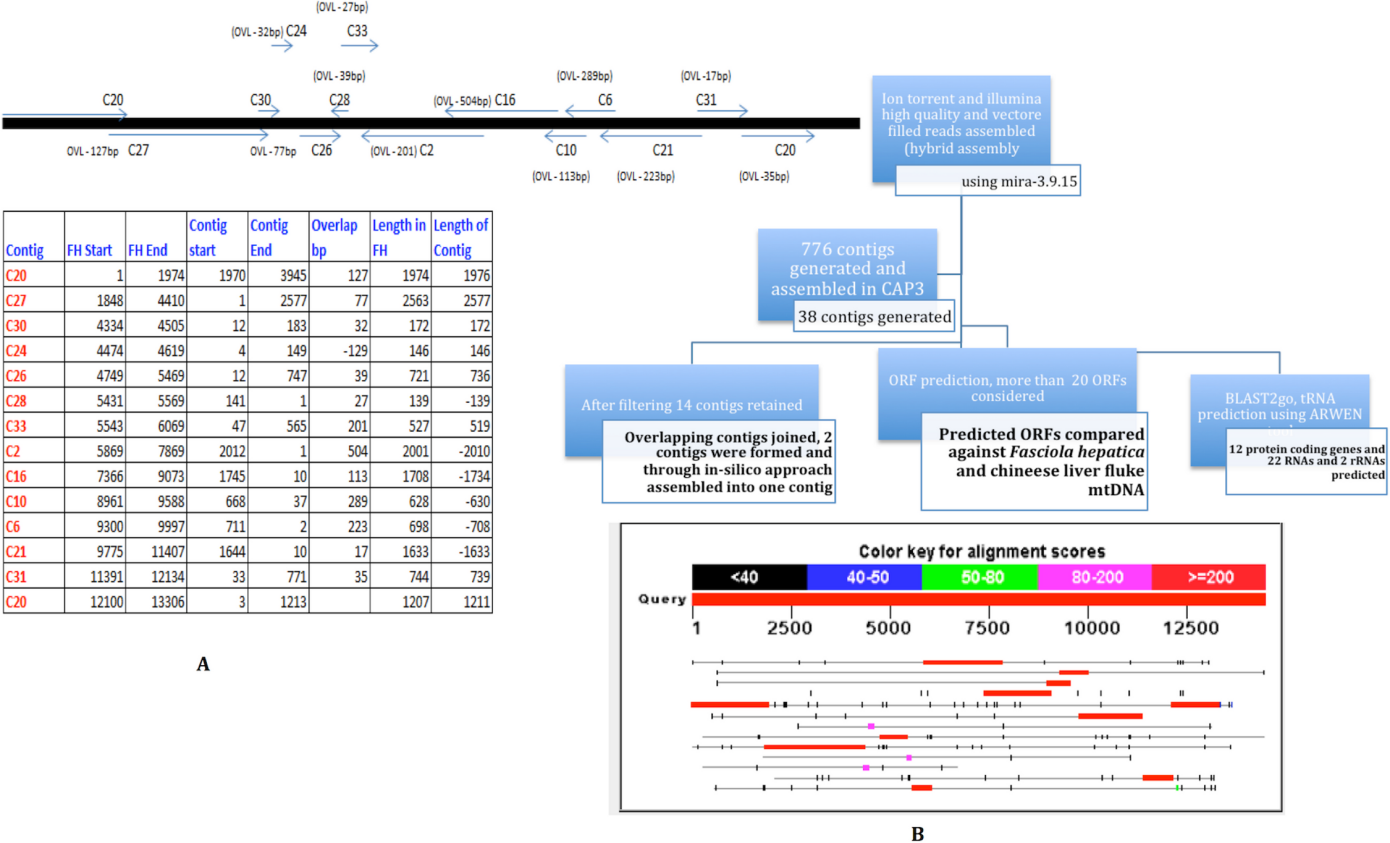
Strategy for MIRA and CAP3 Assembly for mtDNA NGS data. Ion Torrent and Illumina High quality and vector filtered reads assembled (hybrid assembly) using mira-3.9.15. 776 contigs were generated from the hybrid assembly. All the 776 contigs were fed in CAP3 assembler. Post filtering 14 contigs were retained. From 14 contigs overlapping contigs were joined and 2 contigs were formed, which were finally joined as one with the addition of couple of Ns. Predicted ORFs were compared against *F. hepatica* coding regions. Region 1 is a ∼500 nt overlapping region between C2 and C16. Region 2 was sequenced using one primer in C24 and the second primer in C26.

The pooled PCR products were sheared to smaller sizes using Bioruptor. One of each of the Ion Torrent and Illumina library was constructed per manufacturers’ protocols. Briefly, PCR products were sonicated, adapter ligated and amplified for x cycles to generate a library. The libraries were sequenced to generate 14k reads of an average of 150 nt SE reads on Ion Torrent, and 1.3 million reads of 72 nt SE reads on Illumina GAIIx. High quality and vector filtered reads from Ion Torrent and Illumina sequencing were assembled (hybrid-assembly) using Mira-3.9.15 (http://sourceforge.net/apps/mediawiki/mira-assembler). The hybrid assembly generated 776 contigs. All 776 contigs were then used as input for CAP3 assembler which generated 38 contigs. The contigs were further filtered to remove short and duplicate contigs. Finally, only 14 contigs were retained and ORF prediction was carried out using ORF Finder (Open Reading Frame Finder) (http://www.ncbi.nlm.nih.gov/gorf/gorf.html). The schematic outline of the assembly is depicted in Fig. 2.

A manual examination of the 14 contigs revealed overlaps amongst all of them (except C30) (Fig. 2) and in collinear arrangement when compared with the *F. hepatica* mitochondrial sequence. The 14 contigs were manually joined wherever overlaps (minimum overlap >5) were found and that resulted in two individual contigs which, in turn, were assembled into one single contig with the addition of a couple of Ns. To resolve the remaining gaps between the two contigs as well as to confirm the assembly both the regions were amplified and Sanger sequenced. The Sanger sequencing was carried out by designing two primers for both the contigs flanking the Ns to resolve this gap and to verify the assembly as well as closure of the gap that was remaining after joining the contigs manually. The Sanger data in two regions was used to replace the NGS assembly-derived data to refine the assembly and obtain one single contig with no gaps. Region 1 was a ∼500 nt overlapping region between C2 and C16. Region 2 was sequenced using one primer in C24 and the second primer in C26. Considering the finished mitochondrial genome, i.e., from position 1 to 14118, two primer pairs were designed as detailed below:

Set 1: fw primer position # 7395–7414 (Length = 20)
FORWARD PRIMER: TGGTTATTCTGGTTGGGGAG
rev primer position # 8137–8159 (Length = 23)
REVERSE PRIMER: AACCCTCCTATAAGAACCCAAAG (RC = ) CTTTGGGTTCTTATAGGAGGGTT

**Figure 3:**
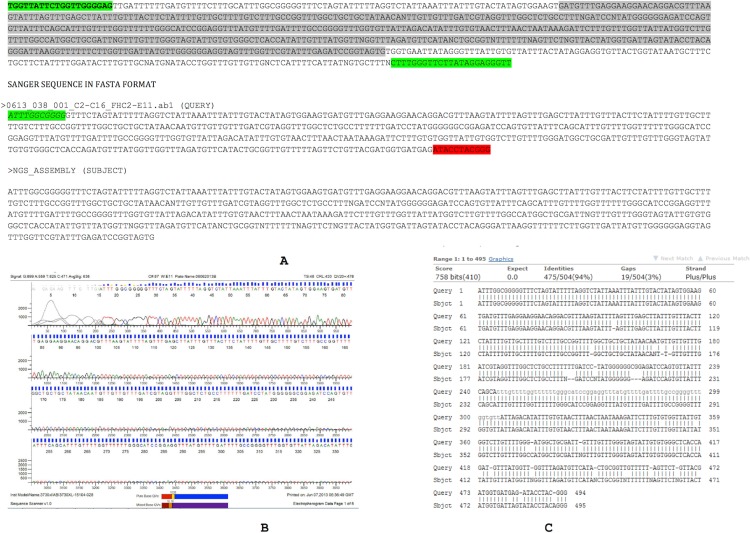
Assembly confirmation of the ∼500 nucleotide region between C2–C16. Primers spanning the 500 bp overlap junction between contig 2 and contig 16 are marked in green. Sanger sequenced region (query) and NGS assembly (subject) were aligned with 94% identity with strong supportive E-values (0.0). Twenty nine out of 494 positions showed discordance between the Sanger sequencing and NGS-derived sequencing consisting of 19 gaps and 10 mismatches that may be introduced by either sequencing chemistry (e.g., homopolymeric stretches in Ion Torrent) or an assembly artifact (e.g., Ns).

The Sanger sequence data and NGS assembly aligned to each other with 94% identity. Twenty-nine out of 494 positions showed discordance between the Sanger sequencing and NGS-derived sequencing for this region (Fig. 3). These discordances consist of 19 gaps and 10 mismatches that can be introduced by either the sequencing chemistry (for e.g., homopolymeric stretches in Ion Torrent) or an assembly artifact (e.g., Ns). Overall, the Sanger sequencing confirmed the assembly pipeline and also corrected errors that are commonly observed in NGS pipelines.

Set 2: fw primer position # 4634–4655 (Length = 22),
FORWARD PRIMER: TAGGGTTATTGGTGTTAACCGG
reverse primer position #4961–4937 (Length = 25)
REVERSE PRIMER: CAACAAACCAACAACTATACATCCC
REV PRIMER RC:- GGGATGTATAGTTGTTGGTTTGTTG

**Figure 4:**
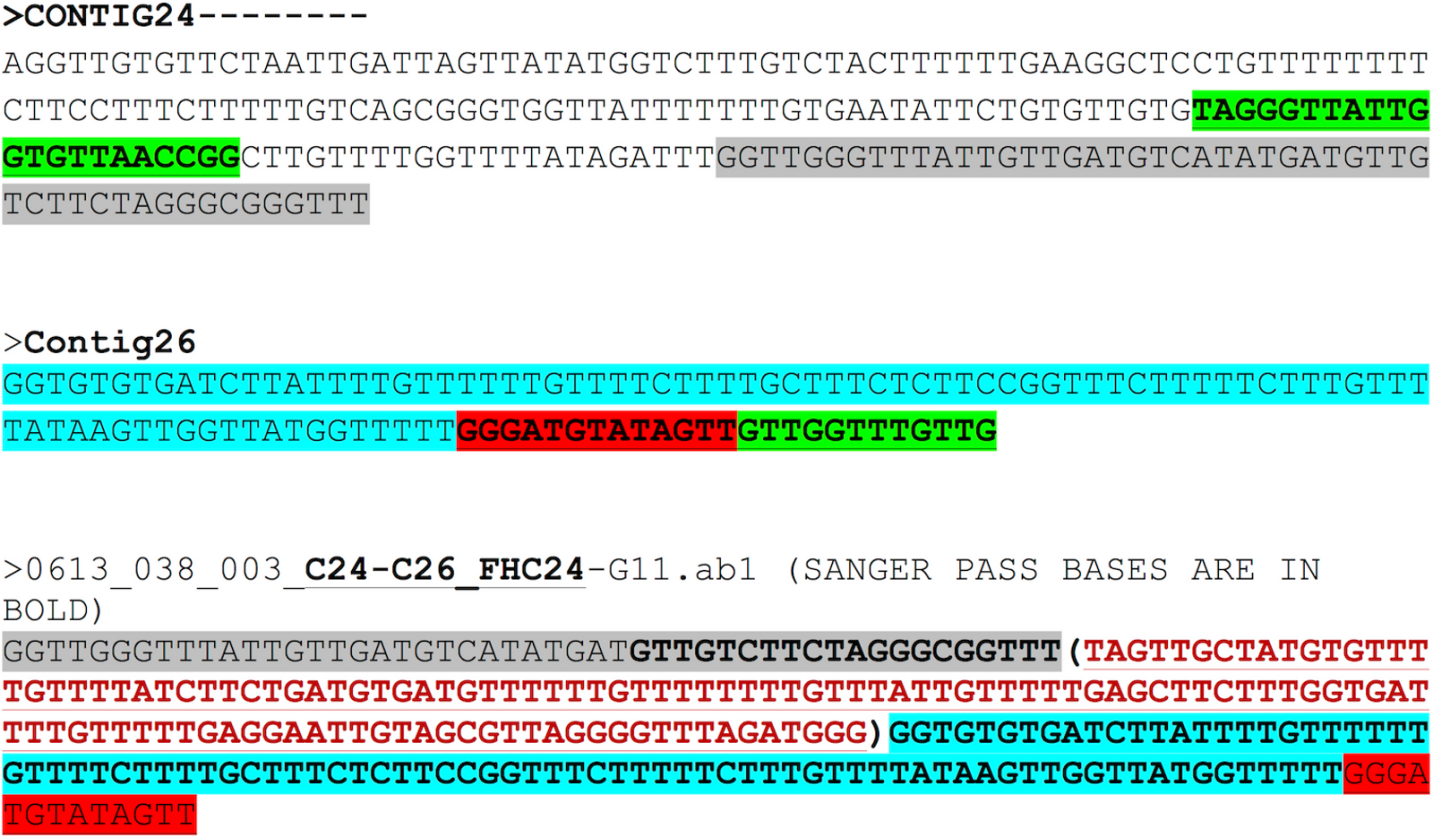
Assembly confirmation for the C24–C26 region. Region between contigs C24 and C26 showing no overlap regions. Forward primer is 94 bp inward from the junction on C24 and the reverse primer 112 bp outward from the junction on C26. The bases in brown colour in brackets are those that fill the gap between C24 and C26.

The region between contigs C24 and C26 did not show any overlap. The forward primer was 94 bp inward from the junction on C24 and the reverse primer was 112 bp outward from the junction on C26. The expected region based on assembly for contigs 24 and 26 and the Sanger results are shown in Fig. 4. The bases in brown colour within brackets are the bases that fill the gap between C24 and C26. Sanger sequencing of the region between C24 and C26 enabled gap-filling of a region that was not sequenced/assembled by the NGS approach and enabled assembly of the mitochondrial genome into one single draft genome.

**Figure 5:**
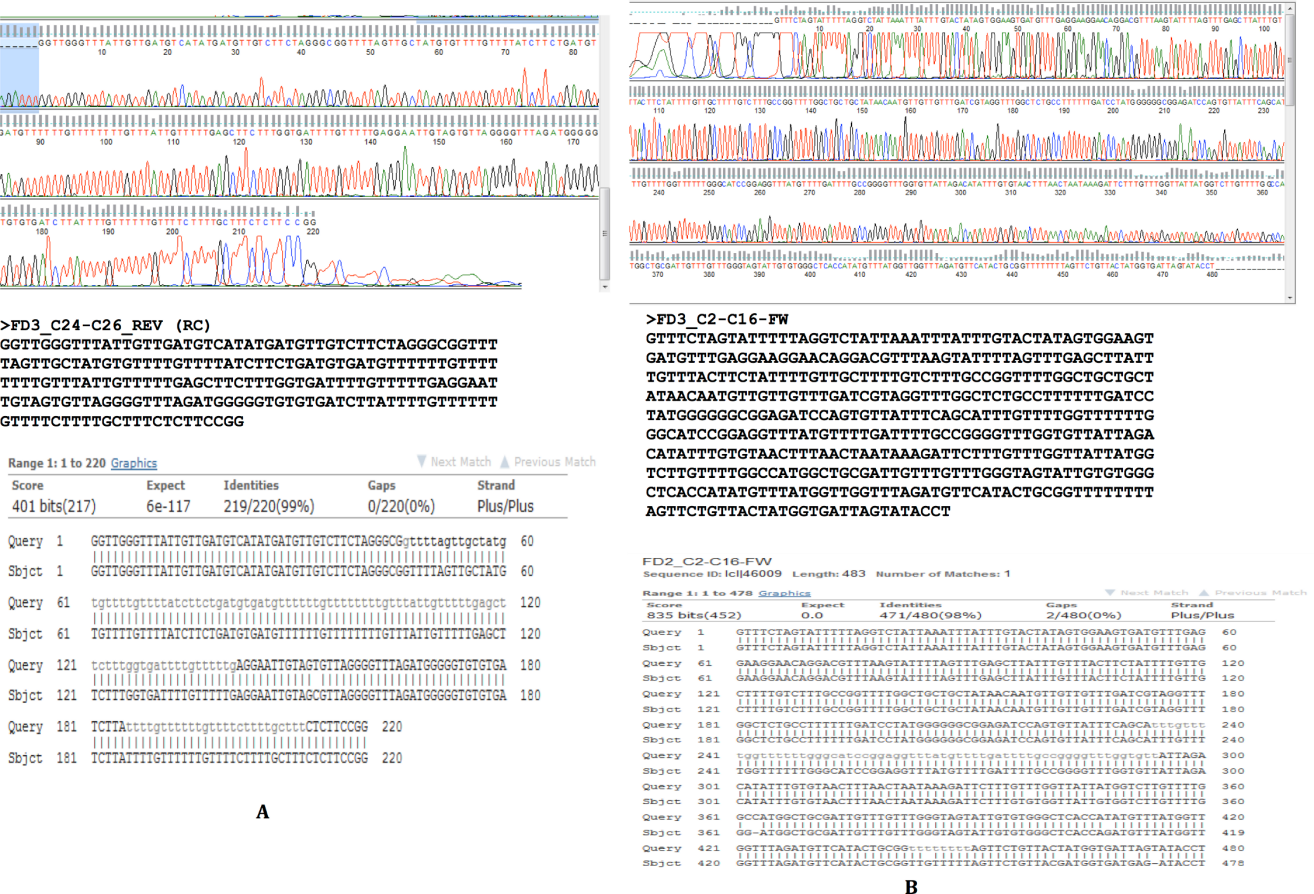
Sanger sequencing confirmatory results for FD2 and FD3 replicate samples. Two separate regions from two independent biological samples sequenced by Sanger methods showing 98–99% identity between samples FD2 (subject) and FD3 (query) in the regions C2–C16 and C24–C26.

To confirm our findings reported herein, whole genomic DNA from an independent *F. buski* sample replicate (Sample FD3) was used and Sanger sequencing was performed on two separate regions (Sample FD3-Region C24–C26 and Sample FD3-Region C2–C16) as described above. The regions from two independent biological sample replicates (FD2 and FD3) by Sanger sequencing exhibited 98–99% identity and thus validated our results (Fig. 5).

The data pertaining to this study is available in the National Centre for Biotechnology Information (NCBI) Bioproject database with Accession: PRJNA210017and ID: 210017. The contig assembly files are deposited in NCBI Sequence Read Archive (SRA) with Accession: SRR924085.

### In silico analysis for nucleotide sequence statistics, protein coding genes (PCGs) prediction, annotation and tRNA prediction

Sequences were assembled and edited both manually and using CLC Genome Workbench V.6.02 with comparison to published flatworm genomes. The platyhelminth genetic code (Telford et al., 2000) was used for translation of reading frames. Protein-coding genes were identified by similarity of inferred amino acid sequences to those of other platyhelminth mtDNAs available in GenBank. Boundaries of rRNA genes both large (rrnL) and small (rrnS) were determined by comparing alignments and secondary structures with other known flatworm sequences. The program ARWEN (Laslett & Canbäck, 2008) was used to identify the tRNA genes (trns). To find all tRNAs, searches were modified to find secondary structures occasionally with very low Cove scores (<0.5) and, where necessary, also by restricting searches to find tRNAs lacking DHU arms (using the nematode tRNA option). Nucleotide codon usage for each protein-encoding gene was determined using the program Codon Usage) at http://www.bioinformatics.org/sms2/codon_usage.html. The ORFs and codon usage profiles of PCGs were analyzed. Gene annotation, genome organization, translation initiation, translation termination codons, and the boundaries between protein-coding genes of mt genomes of the two fasciolid flukes were identified based on comparison with mt genomes of other trematodes reported previously (Le et al., 2002). The mtDNA genome of *F. buski* was annotated taking *F. hepatica* as a reference genome using several open source tools viz. Dual Organellar Genome Annotator (DOGMA) (Wyman, Jansen & Boore, 2004), Organellar Genome Retrieval System (OGRe) (Jameson et al., 2003) and Mitozoa database (D’Onorio de Meo et al., 2011). The newly sequenced and assembled *F. buski* mtDNA was sketched with GenomeVX at http://wolfe.ucd.ie/GenomeVx/ with annotation files from DOGMA (Wyman, Jansen & Boore, 2004).

### Phylogenetic analysis

**Figure 6:**
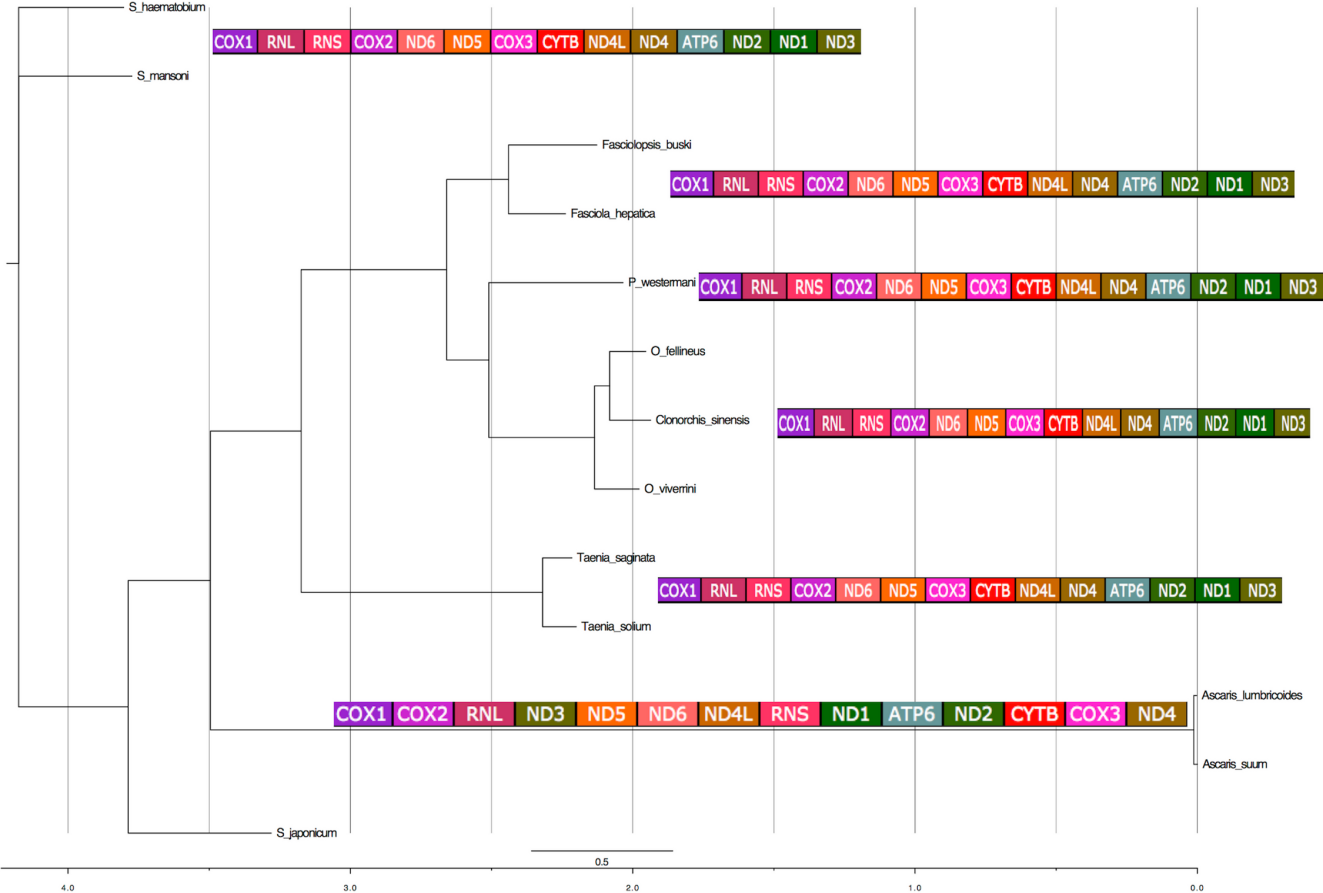
Phylogenetic analysis of the concatenated 12 protein coding genes from the platyhelminth mtDNA. Differences in the gene order in the mitochondrial genomes of parasitic flatworms from the Trematoda and Cestoda and taking Nematoda (Ascaridida) as an outgroup. Phylogenetic analyses of concatenated nucleotide sequence datasets for all 12 PCGs were performed using Bayesian Inference using four MCMC chains and 106 generations, sampled every 1,000 generations. Bayesian posterior probability (BPP) values were determined after discarding the initial 200 trees (the first 2 × 105 generations) as burn-in. Using the phylogeny estimated from the nuclear ribosomal DNA data set, pictograms of full mitochondrial genes are indicated next to the individual leaves of the tree. *x*-axis represents substitution rates per unit.

The 12 PCGs were concatenated and a super matrix was created in Mesquite (Maddison & Maddison, 2001) and run in MrBayes (Ronquist & Huelsenbeck, 2003). Phylogenetic analyses of concatenated nucleotide sequence datasets for all 12 PCGs were performed using Bayesian inference [BI]). MrBayes was executed using four MCMC chains and 106 generations, sampled every 1,000 generations. Each of the 12 genes was treated as a separate unlinked data partition. Bayesian posterior probability (BPP) values were determined after discarding the initial 200 trees (the first 2 × 105 generations) as burn-in. Using the phylogeny estimated from the nuclear ribosomal DNA data set, pictograms of full mitochondrial genes indicating the gene order were aligned next to the individual ‘leaves’ of the tree (Fig. 6).

## Results and Discussion

### Gene contents and organization

**Figure 7:**
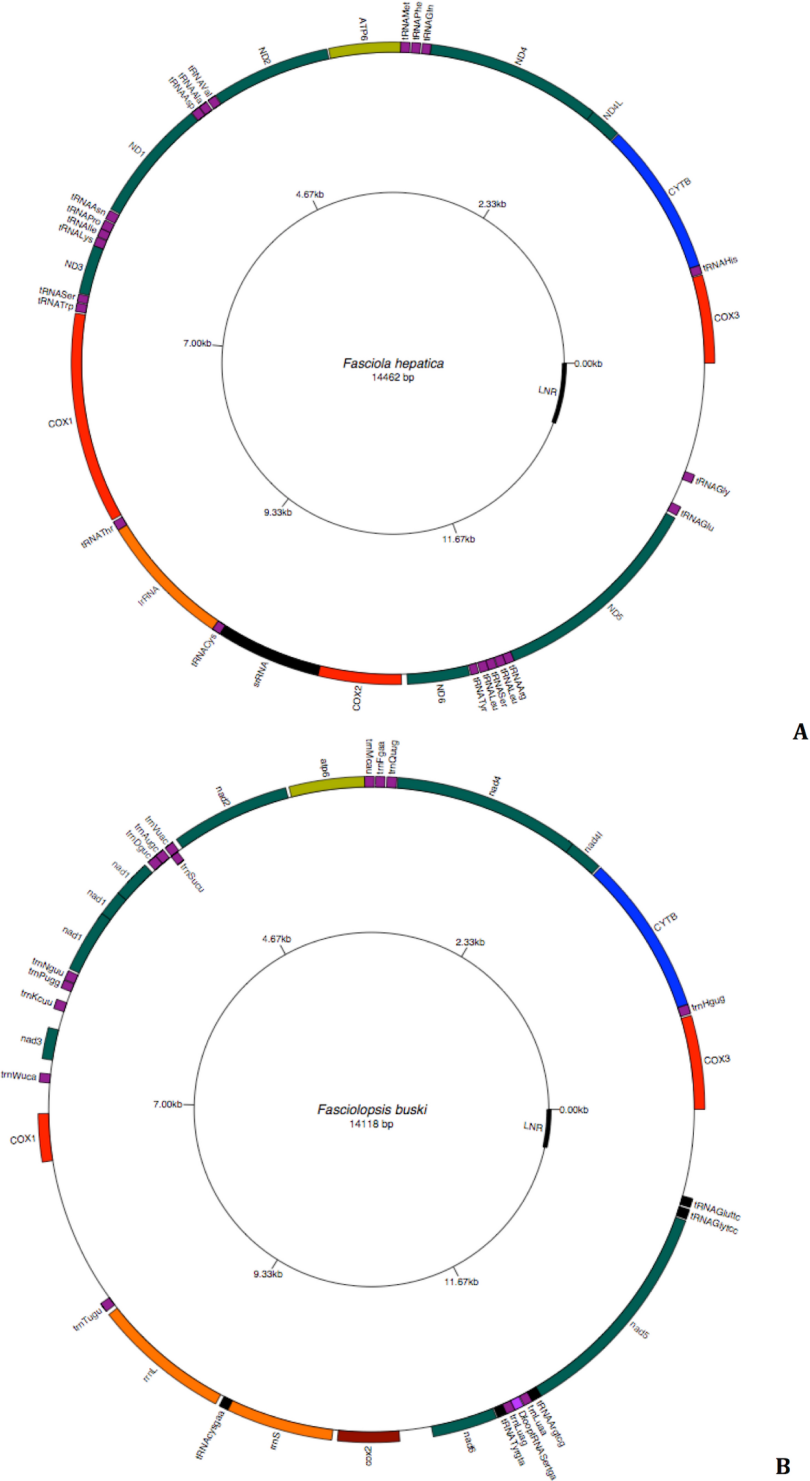
Circular genome map of *Fasciola hepatica* and *Fasciolopsis buski*. The manual and in silico annotations with appropriate regions for *F. buski* (7A) and annotated GenBank flat file for *F. hepatica* (7B) were drawn into a circular graph in GenomeVX depicting the 12 PCGs and 22tRNAs.

The intestinal fluke *F. buski* has a mt genome typical of most platyhelminths (Fig. 7A). The circular genome consists of 14118 nt bp and is almost similar to that of *Fasciola hepatica* (Fig. 7B). The 12 protein-coding genes fall into the following categories: nicotinamide dehydrogenase complex (nad1–nad6 and nad4L subunits); cytochrome c oxidase complex (cox1–cox3 subunits); cytochrome b (cob) and adenosine triphosphatase subunit 6 (atp6). Two genes encoding ribosomal RNA subunits are present: the large subunit (rrnL or 16S) and small subunit (rrnS or 12S), which are separated by trnC, encoding the transfer RNA (tRNA) for cysteine. As in other mt genomes, there are 22 tRNA genes, denoted in the figure by the one-letter code for the amino acid they encode. Leu and Ser are each specified by two different tRNAs, reflecting the number and base composition of the relevant codons. As in other flatworms, all genes are transcribed in the same direction (Fig. 7). Genes lack introns and are usually adjacent to one another or separated by only a few nucleotides. However, some genes overlap, most notably nad4, nad4L and with regions of the long non coding region, which is almost 500 nt length.

### Nucleotide composition and codon usage

Invertebrate mt genomes tend to be AT-rich (Malakhov, 1994), which is a notable feature in PCGs of several parasitic flatworms. However, nucleotide composition is not uniform among the species (Table 2). Values for >70% AT are seen in all *Schistosoma* spp. except for *S. mansoni* (68.7%), whereas *F. buski* and *Fasciola hepatica* are 60% AT rich and *Paragonimus westermani*, only 50% AT rich. Cytosine is poorly represented in *F. buski*. The annotation and nucleotide sequence statistics are enumerated in Tables 2–5. The gene content and arrangement are identical to those of *F. hepatica*. ATG and GTG are used as the start-codons and TAG and TAA, the stop-codons.

**Table 2:** Mitochondrial DNA nucleotide sequence statistics information of Platyhelminths.

Sequence type	DNA	DNA	DNA	DNA	DNA	DNA	DNA	DNA	DNA	DNA	DNA	DNA	DNA
Length	14,118 bp circular	14,462 bp circular	14,965 bp circular	14,277 bp circular	13,510 bp	13,875 bp circular	15,003 bp circular	14,415 bp circular	14,085 bp circular	13,670 bp circular	13,709 bp circular	14,281 bp circular	14,284 bp circular
Organism name	*Fasciolopsis* *buski*	*Fasciola* *hepatica*	*Paragonimus* *westermani*	*Opisthorchis* *felineus*	*Opisthorchis* *viverrini*	*Clonorchis* *sinensis*	*Schistosoma* *haematobium*	*Schistosoma* *mansoni*	*Schistosoma* *japonicum*	*Taenia* *saginata*	*Taenia* *solium*	*Ascaris* *lumbricoides*	*Ascaris* *suum*
Accession	Submitted to GenBank	NC_002546	NC_002354	EU921260	JF739555	FJ381664	NC_008074	NC_002545	NC_002544	NC_009938	NC_004022	JN801161	NC_001327
Modification date	submitted	01-FEB- 2010	01-FEB- 2010	18-AUG- 2010	05-APR- 2012	01-JUL- 2010	14-APR- 2009	14-APR- 2009	01-FEB- 2010	14-APR- 2009	01-FEB- 2010	01-DEC- 2011	11-MAR- 2010
Weight (single-stranded)	4396.507	4,499.496 kDa	4,652.101 kDa	4,437.683 kDa	4,197.397 kDa	4,311.834 kDa	4,658.966 kDa	4,482.165 kDa	4,371.002 kDa	4,242.425 kDa	4,251.992 kDa	4,428.619 kDa	4,429.981 kDa
Weight (double-stranded)	8721.667	8,934.244 kDa	9,246.535 kDa	8,820.283 kDa	8,346.532 kDa	8,571.888 kDa	9,266.949 kDa	8,904.302 kDa	8,700.11 kDa	8,443.711 kDa	8,467.723 kDa	8,443.711 kDa	8,822.899 kDa
**Annotation table**													
Feature type	Count	Count	Count	Count	Count	Count	Count	Count	Count	Count		Count	Count
CDS	12	12	12	12	12	12	12	12	12	12	12	12	12
Gene	12	12	12	12	12	12	12	12	12	12	12	12	12
Misc. feature	1	1	-	-	-	-		1	1	-	-	1	2
rRNA	2	2	1	2	2	2	2	2	2	2	2	2	2
tRNA	22	22	23	22	20	22	22	23	23	22	22	22	22

**Table 3:** Atomic composition and nucleotide distribution table of *Fasciolopsis buski* mtDNA. Ambiguous residues are omitted in atom counts.

Atom	Count	Frequency	Nucleotide	Count	Frequency
**As single stranded**			Adenine (A)	2509	0.178
Hydrogen (H)	174924	0.376	Cytosine (C)	1281	0.091
Carbon (C)	139209	0.299	Guanine (G)	3925	0.278
Nitrogen (N)	48681	0.105	Thymine (T)	6334	0.449
Oxygen (O)	88120	0.19	Purine (R)	0	0
Phosphorus (P)	14049	0.03	Pyrimidine (Y)	0	0
**As double stranded**			Adenine or cytosine (M)	0	0
Hydrogen (H)	346023	0.375	Guanine or thymine (K)	0	0
Carbon (C)	275774	0.299	Cytosine or guanine (S)	0	0
Nitrogen (N)	103549	0.112	Adenine or thymine (W)	0	0
Oxygen (O)	168590	0.183	Not adenine (B)	0	0
Phosphorus (P)	28098	0.03	Not cytosine (D)	0	0
			Not guanine (H)	0	0
			Not thymine (V)	0	0
			Any nucleotide (N)	69	0.005
			C + G	5206	0.369
			A + T	8843	0.626

**Table 4:** Codon usage for *F. buski* mtDNA genome.

AmAcid	Codon	Number	/1000	Fraction
Ala	GCG	22.00	4.74	0.20
Ala	GCA	26.00	5.60	0.24
Ala	GCT	46.00	9.91	0.43
Ala	GCC	14.00	3.02	0.13
Cys	TGT	239.00	51.49	0.80
Cys	TGC	58.00	12.49	0.20
Asp	GAT	90.00	19.39	0.85
Asp	GAC	16.00	3.45	0.15
Glu	GAG	63.00	13.57	0.68
Glu	GAA	30.00	6.46	0.32
Phe	TTT	442.00	95.22	0.85
Phe	TTC	79.00	17.02	0.15
Gly	GGG	119.00	25.64	0.28
Gly	GGA	67.00	14.43	0.16
Gly	GGT	201.00	43.30	0.48
Gly	GGC	31.00	6.68	0.07
His	CAT	19.00	4.09	0.73
His	CAC	7.00	1.51	0.27
Ile	ATT	168.00	36.19	0.86
Ile	ATC	28.00	6.03	0.14
Lys	AAG	42.00	9.05	1.00
Leu	TTG	263.00	56.66	0.36
Leu	TTA	193.00	41.58	0.27
Leu	CTG	70.00	15.08	0.10
Leu	CTA	53.00	11.42	0.07
Leu	CTT	117.00	25.20	0.16
Leu	CTC	26.00	5.60	0.04
Met	ATG	101.00	21.76	0.58
Met	ATA	72.00	15.51	0.42
Asn	AAA	39.00	8.40	0.34
Asn	AAT	64.00	13.79	0.56
Asn	AAC	11.00	2.37	0.10
Pro	CCG	16.00	3.45	0.27
Pro	CCA	10.00	2.15	0.17
Pro	CCT	27.00	5.82	0.45
Pro	CCC	7.00	1.51	0.12
Gln	CAG	19.00	4.09	0.63
Gln	CAA	11.00	2.37	0.37
Arg	CGG	34.00	7.32	0.33
Arg	CGA	15.00	3.23	0.14
Arg	CGT	44.00	9.48	0.42
Arg	CGC	11.00	2.37	0.11
Ser	AGG	100.00	21.54	0.25
Ser	AGA	49.00	10.56	0.12
Ser	AGT	88.00	18.96	0.22
Ser	AGC	17.00	3.66	0.04
Ser	TCG	30.00	6.46	0.07
Ser	TCA	25.00	5.39	0.06
Ser	TCT	69.00	14.86	0.17
Ser	TCC	28.00	6.03	0.07
Thr	ACG	7.00	1.51	0.14
Thr	ACA	14.00	3.02	0.27
Thr	ACT	19.00	4.09	0.37
Thr	ACC	11.00	2.37	0.22
Val	GTG	114.00	24.56	0.22
Val	GTA	95.00	20.47	0.19
Val	GTT	270.00	58.16	0.53
Val	GTC	34.00	7.32	0.07
Trp	TGG	174.00	37.48	0.60
Trp	TGA	115.00	24.77	0.40
Tyr	TAT	160.00	34.47	0.83
Tyr	TAC	32.00	6.89	0.17
End	TAG	118.00	25.42	0.65
End	TAA	63.00	13.57	0.35
**Counts of di-nucleotides in *F. buski* mtDNA**				
**1.pos∖2.pos**	**A**	**C**	**G**	**T**
A	467	191	766	1072
C	217	204	262	593
G	656	347	1263	1645
T	1158	530	1619	2997
**Frequency of di-nucleotides in *F. buski* mtDNA**				
**1.pos∖2.pos**	**A**	**C**	**G**	**T**
A	0.033	0.014	0.055	0.077
C	0.016	0.015	0.019	0.042
G	0.047	0.025	0.09	0.118
T	0.083	0.038	0.116	0.214

**Table 5:** mtDNA annotation of *F. buski* and comparison with *Fasciola hepatica*.

	Gene	Length in *F. hepatica*	Gene prediction length in *F. buski*	% of *F. hepatica* CDS covered in *F. buski*
1	nad3	118	97	82.20
2	nad2	288	257	89.24
3	cox1	510	470	92.16
4	nad1	300	278	92.67
5	cox2	200	194	97.00
6	cox3	213	210	98.59
7	nad5	522	515	98.66
8	cob	370	366	98.92
9	nad6	150	149	99.33
10	nad4L	90	90	100.00
11	nad4	423	423	100.00
12	atp6	172	172	100.00

**Table 6:** Transfer RNA (tRNA) annotations of the *Fasciolopsis buski* mtDNA.

Contig start	Contig end	Length	tRNA ID	Codon	GC %	Single letter symbol
657	722	66	mtRNA-His	GUG	30.3	H
3358	3424	67	mtRNA-Gln	UUG	35.8	Q
3438	3501	64	mtRNA-Phe	GAA	39.1	F
3511	3576	66	mtRNA-Met	CAU	40.9	M
5086	5150	65	mtRNA-Val	UAC	32.3	V
5168	5233	66	mtRNA-Ala	UGC	40.9	A
5233	5300	68	mtRNA-Asp	GUC	38.2	D
6213	6277	65	mtRNA-Asn	GUU	41.5	N
6282	6339	58	TV-loop mtRNA-Pro	UGG	31	P
6356	6418	63	mtRNA-Ile	GAU	47.6	I
6421	6487	67	mtRNA-Lys	CUU	38.8	K
6859	6919	61	D-loop mtRNA-Ser	GCU	41	S1
6932	6994	63	mtRNA-Trp	UCA	34.9	W
8577	8641	65	mtRNA-Thr	UGU	30.8	T
9642	9707	66	mtRNA-Cys	GCA	50	C
11581	11646	66	mtRNA-Tyr	GUA	39.4	Y
11645	11709	65	mtRNA-Leu	UAG	49.2	L1
11708	11772	65	D-loop mtRNA-Ser	UGA	36.9	S2
11778	11841	64	mtRNA-Leu	UAA	37.5	L2
11839	11910	72	mtRNA-Arg	UCG	37.5	R
13483	13551	69	mtRNA-Gly	UCC	30.4	G
13565	13628	64	mtRNA-Glu	UUC	43.8	Q

**Figure 8:**
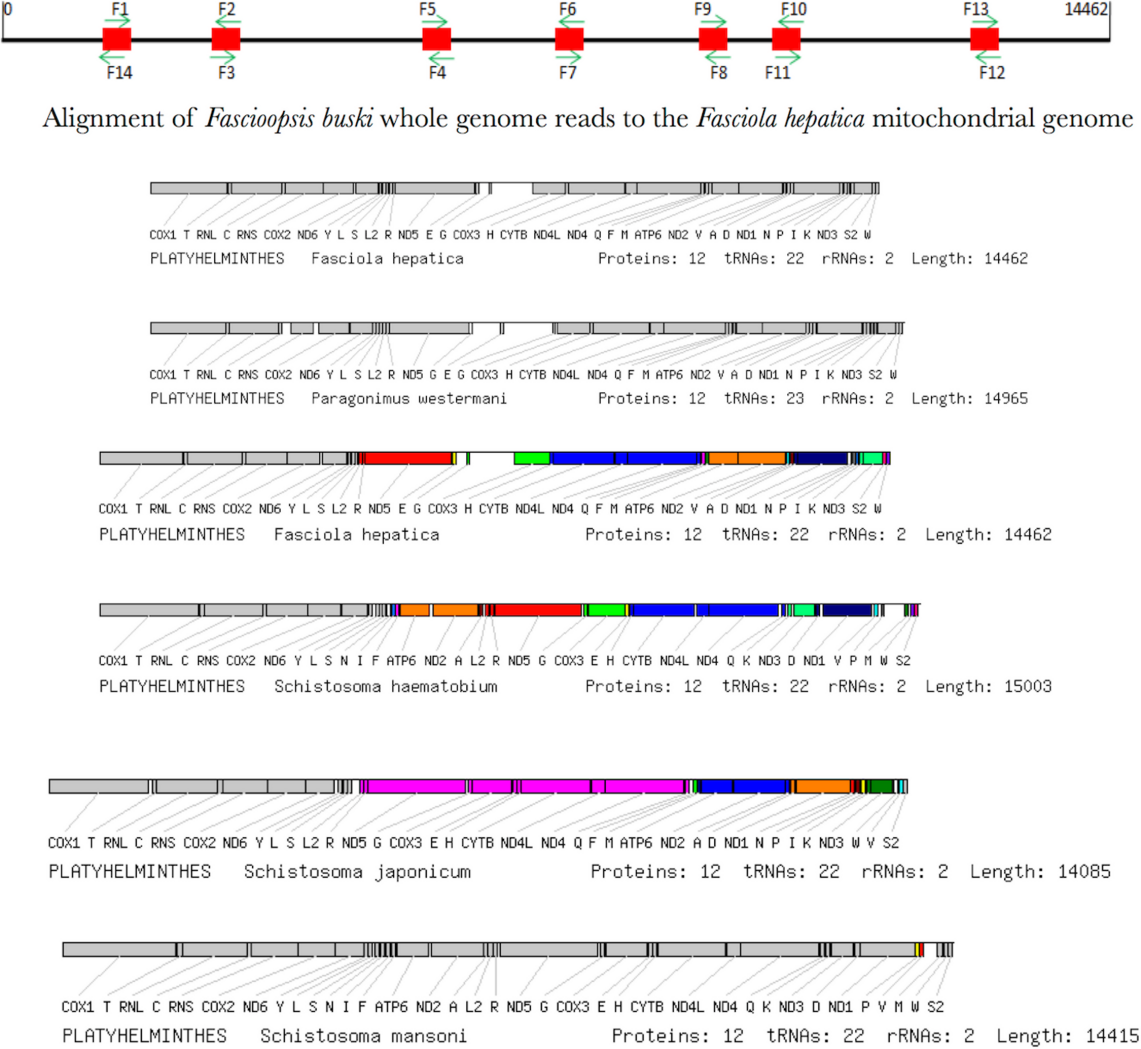
Synteny map of the representative species for the platyhelminth mtDNA. A comparative synteny for all the 12 protein coding genes and 22/23 tRNAs for the representative platyhelminth parasites (Schistosoma spp, *F. buski*, *Fasciola hepatica*, *Paragonimus westermani*).

Among species considerable differences in base composition in PCGs are reflected in differences in the protein sequences. However, the redundancy in the genetic code provides a means by which a mt genome could theoretically compensate for base-composition bias. Increased use of abundant bases in the (largely redundant) third codon position accounts for the fact that base composition bias would be less marked in the first and second codon positions. A phylogenetic tree was computed concatenating all the annotated 12 PCGs that completely accounted for the platyhelminth phylogeny with the representative species (Fig. 8). *F. buski* came in the same clade with *F. hepatica* while *Ascaris* species formed the outgroup. The outgroup *Ascaris lumbricoides* displayed a different gene order that was aligned adjacent to the phylogenetic leaf nodes (Fig. 8).

### Transfer and ribosomal RNA genes

**Figure 9:**
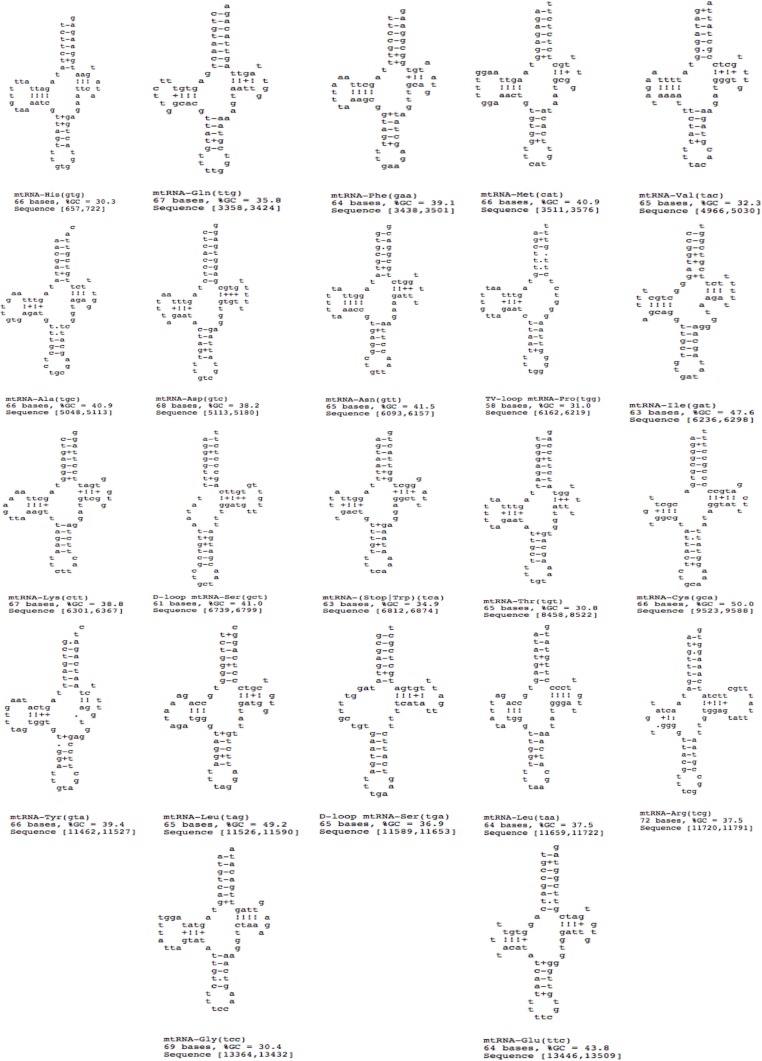
22 tRNA secondary structures predicted using ARWEN.

A total of 22 tRNAs were inferred along with structures (Fig. 9). The complete annotation along with their GC percentage is shown in Table 6. tRNA-Leu had the highest GC composition and the length varied between 60–70 nt bases. The tRNA genes generally resemble those of other invertebrates. A standard cloverleaf structure was inferred for most of the tRNAs. Exceptions include tRNA(S) in which the paired dihydrouridine (DHU) arm is missing as usual in all parasitic flatworm species (also seen in some other metazoans) and also tRNA(A) in which the paired DHU-arm is missing in cestodes but not in trematodes (and not usually in other metazoans) and hence, was also seen in *F. buski*. Structures for tRNA(C) vary somewhat among the parasitic flatworms. A paired DHU-arm is present in *F. buski*, which is not seen in *Schistosoma mekongi* and cestodes. A comparative synteny for all the 12 protein coding genes and 22/23 tRNAs for the representative platyhelminth parasites can be seen across all the species under study (Fig. 8).

## Conclusions

Although mt genomes of only a few parasitic flatworms have been sequenced, some general points can be made. The mtDNA of *F. buski* did not exhibit any surprising gene order composition or their organization relative to other invertebrates. As usual atp8 was absent, which is not without a precedent among invertebrates. Some typical secondary structures were inferred for some tRNA genes. Again, however, mt tRNA genes are less conserved in metazoans as compared to their nuclear counterparts. Gene order is similar or identical among most of the flatworms investigated, which might be expected for a taxon at this level of taxonomic heirarchy. In conclusion, the complete mtDNA sequences of *F. buski* will add to the knowledge of the trematode mitochondrial genomics and will aid in phylogenetic studies of the family Fasciolidae.

## Additional Information and Declarations

### DNA Deposition

The following information was supplied regarding the deposition of DNA sequences: National Centre for Biotechnology Information (NCBI) Bioproject database: PRJNA210017 and ID: 210017. NCBI Sequence Read Archive (SRA): SRR924085.
